# Goal-based outcomes of hospitalisation of older adults are predicted by gender, confidence, quality of life and type of goals

**DOI:** 10.1007/s41999-022-00698-2

**Published:** 2022-10-07

**Authors:** Maria Johanna van der Kluit, Sanne Tent, Geke J. Dijkstra, Sophia E. de Rooij

**Affiliations:** 1 University of Groningen, University Medical Center Groningen, University Center for Geriatric Medicine, Hanzeplein 1, 9700 RB Groningen, Netherlands; 2 Research Group Living, Wellbeing and Care for Older People, NHL Stenden University of Applied Sciences, Leeuwarden, Netherlands; 3 Department of Health Sciences, Applied Health Research, University of Groningen, University Medical Center Groningen, Groningen, Netherlands; 4 Amstelland Hospital, Amstelveen, Netherlands

**Keywords:** PROM, Hospitalisation, Confidence, Hope, Goal-based care

## Abstract

**Purpose:**

Although patient-reported outcome measures (PROMs) might reflect relevant outcomes from patient perspective, they do not always reflect what the individual patient finds important. Our objectives were to assess which PROM was best suited to evaluate patient-relevant outcomes of hospitalisation and to assess which factors predicted this PROM.

**Methods:**

A longitudinal study was conducted among hospitalised older patients. Three PROMs were compared with the anchor question ‘How much have you benefited from the admission?’: a general quality of life measure: EQ-5D; a measure of daily functioning: Katz-15 and a goal-based measure: achievement of self-defined goals. Predictors were examined using logistic regression analyses.

**Results:**

We had 185 cases with baseline and follow-up. Accomplishment of self-defined goals showed a large correlation with the anchor question, whereas EQ-5D and Katz-15 showed no significant correlations. The final regression model had four predictors: being man, having higher confidence in goal achievement and good/excellent quality of life increased the odds for goal accomplishment, while having goals in the category alleviating complaints reduced the odds.

**Conclusion:**

Accomplishment of individual goals represented the benefit experienced by participants best. Subjective indicators of health and functioning are better predictors of goal accomplishment than objective ones. According to participant experience, the hospital appeared successful in managing disease-specific problems, but less successful in ameliorating complaints. Medical decision-making should not only be based on medical indicators, but the input of the patient is at least as important. Quality of life, goals and confidence should be discussed. More attention is needed for symptom experience.

**Supplementary Information:**

The online version contains supplementary material available at 10.1007/s41999-022-00698-2.

## Key summary points

**Aim:**

To assess which PROM was best suited to evaluate patient-relevant outcomes of hospitalisation and to assess which factors predicted this PROM.

**Findings:**

Accomplishment of self-defined goals represented the benefit experienced by hospitalisation best, whereas EQ-5D and Katz-15 showed no significant correlations. Subjective indicators of health and functioning are better predictors of goal accomplishment than objective.

**Message:**

Medical decision-making should not only be based on objective medical indicators, but also on subjective indicators such as quality of life, symptom experience, goals and confidence in goal achievement.

**Supplementary Information:**

The online version contains supplementary material available at 10.1007/s41999-022-00698-2.

## Background

Outcomes of hospitalisation are complex to measure and predict. Quantitative outcomes can be described with objective or subjective measures. Examples of objective, often administrative measures are length of stay, mortality, test results or clinical performance indicators. Subjective measures are patient-reported outcomes (PROs), which are dependent on the judgement of the individual patient; examples are symptom burden, functional status and quality of life [1–3]. Although PROs might reflect relevant outcomes from patient perspective better than objective outcomes [2], they do not always reflect what patients find important, since patient involvement in the development of instruments is rare [4]. But even when patients are involved in the development of patient-reported outcome measures (PROMs), these often only reflect the priorities of the overall patient population and do not take into account the individual, while personal and not average outcomes on a group’s level are considered important to individual patients and preferred outcomes differ per individual [5, 6].

A large part of the hospital population consists of older patients [7]. Taking into account individual priorities is even more important for older patients, as they often suffer from multiple chronic diseases. For these patients, a shift is recommended from disease-oriented towards goal-oriented care [8, 9].

To measure individualised meaningful PROMs, the Patient Benefit Assessment Scale for Hospitalised Older Patients (P-BAS HOP) was developed, which was further refined and transformed into the P-BAS Picture version (P-BAS-P) [10, 11]. Both instruments were designed to select and assess the importance of various predefined personal goals during hospitalisation and to evaluate the achievement of goals during follow-up. Based on these data an individual Patient Benefit Index (PBI) was computed, which is an overall value reflecting the achievement of the set goals weighted by their importance. Unfortunately, there were problems with the reliability, validity and responsivity of the instruments [11, 12], causing a debate whether a goal-based approach is the optimal approach to assess individual patient-relevant outcomes of hospitalisation.

In the last years, much attention is paid to functional outcomes after hospitalisation, as around 30% of acute hospitalised older patients experience hospital-associated disability (HAD), which is defined as a loss of independence in activities of daily living (ADL) following hospitalisation [13, 14]. Several factors associated with functional outcomes after hospitalisation are described, including personal characteristics, functioning, health, and characteristics of admission. [15–19]. However, unknown is what the relationship of these factors is with the PROM most relevant to the individual patient.

Therefore, the aims of this study were (1) to assess which PROM is best suited to evaluate the individual patient’s relevant outcomes of hospitalisation and (2) to assess which factors predict this best suitable outcome measure.

## Methods

### Design and population

This longitudinal study was performed among consecutive hospitalised older patients. The first face-to-face standardised interview took place within the first 4 days of hospitalisation. The follow-up interview was performed 3 months after discharge by telephone.

Because we wanted a broad group of older patients, eligible participants were 70 years and older; either had a planned or unplanned hospital admission on medical or surgical wards of a university teaching hospital in the Netherlands, were able to speak and understand Dutch and were without cognitive impairment. Every weekday, all consecutive patients admitted to medical and surgical wards were selected in the hospital administration according to age and admission date by a trained research assistant. Then the staff nurses were contacted to verify further inclusion criteria. Patients were approached by a trained research assistant and gave signed informed consent.

The Medical Ethics Research Committee of the UMCG (file number M16.192615) confirmed that the Medical Research Involving Human Subjects Act did not apply to the research project. Official approval by the committee was therefore not required.

### PROMs

We compared the following PROMs: a general quality of life measure: EQ-5D; a measure of daily functioning: Katz-15 scale and a goal-based measure: achievement of self-defined goals. These outcomes were compared with the anchor question: ‘How much have you benefited from the admission?’ with the following answer options: not at all, a little bit, somewhat, much, very much.

#### EQ-5D

The EQ-5D is a standardised, non-disease-specific instrument for describing and valuing health-related quality of life, consisting of five dimensions and a visual analogue scale (VAS) [20]. The dimensions are mobility, self-care, usual activities, pain/discomfort and anxiety/depression, with three answer options each: no problems, some problems and extreme problems. A single index value was generated by aggregating and weighting the five domains, using de Dutch EQ-5D tariff. A value of 1 refers to full health and 0 refers to death, while negative values are possible and refer to health states considered worse than death. The VAS, often referred to as the EuroQol ‘thermometer’, has an end point of 100 points for best imaginable health state and 0 points for worst imaginable health state [20]. Participants filled in the EQ-5D three times: during the baseline interview, to indicate their state 2 weeks prior to hospital admission, as well as the day of interview and during the follow-up interview, to indicate their state at the day of interview. We computed two difference scores: between follow-up and prior to admission; and between follow-up and during admission.

#### Katz-15 scale

The Katz-15 scale consists of 15 items regarding basic activities of daily living (such as the need for help with bathing) and Instrumental Activities of Daily Living (such as shopping) with dichotomous answer options [21]. Participants were asked, during the baseline interview, to indicate their functioning 2 weeks prior to hospital admission, and during the follow-up interview, to indicate their functioning on the day of interview. Items were summed, and a higher score means more dependency. A difference score was computed between the follow-up and situation before admission.

#### Self-defined goals

The participant was asked at baseline: ‘What do you hope to accomplish with this hospitalisation?’ and named up to five goals. At follow-up, goals stated by the participant were repeated whereafter the participant was asked per goal to what extent the goal was accomplished with the answer options: ‘not at all’, ‘somewhat’, ‘moderately’ ‘quite’, or ‘completely’. The answer options were scored on an ordinal scale from 1 (not at all) to 5 (completely). When two or more goals were scored, the mean score was calculated for calculating the correlation with the anchor question.

### Predicting factors

Predicting factors and their operationalisation, selected from literature, are displayed in Table 1. We categorised them into personal characteristics, functioning, health, and characteristics of admission. In addition, we added the characteristics of goals. All goals, defined by participants, were categorised as: wanting to know what the matter is; controlling disease; staying alive; improving condition; alleviating complaints; daily functioning; social functioning; resuming work, hobbies; autonomy [5]. Two researchers (MJvdK, ST) independently categorised the goals and compared results. When a goal could not be categorised into one of the existing categories, it was categorised as ‘other’. Discrepancies were solved by consensus. Since the variety of categories caused low frequencies per category, we combined several categories. Also answer options of diverse other predictors were combined when frequencies were too low for analysis.

**Table 1:** Predicting factors

Factor	Operationalisation
Personal characteristics	
Age	Years
Gender	Male/female
Living situation	Independent/sheltered accommodation/senior home/nursing home
Marital status	Alone/with partner
Health locus of control	Internal/powerful others/chance based of the multidimensional health locus of control scales (MHLC) [22]. We used the Dutch version of the MHLC. A higher score means more belief in that dimension [23]
Baseline quality of life	Single question: ‘How is in general your quality of life?’ Bad/ reasonable/ good/ very good/ excellent. For analysis we combined the options bad/reasonable and very good/excellent
Functioning	
(I)ADL	Katz-15 scale [21]
Cognitive functioning	For months backward test, we used the detailed grading for research as recommended by [24]. For analysis we combined several grades into the scores: cannot reach January/can reach January with errors/completes test without errors
Social functioning	Single question from the 36-item Short Form Survey Instrument (SF-36): question ‘During the past 4 weeks, how much of the time has your physical health or emotional problems interfered with your social activities (like visiting with friends, relatives, etc.)?’ none of the time, a little of the time, some of the time, most of the time, all of the time [25] For analysis we combined the options most of the time/all of the time
Hearing	Single question: ‘Are you able to hear well, with or without hearing aid?’ Yes/no
Mobility	Single question: ‘Were you able to walk outside for five minutes?’ Impossible/only with help of somebody else/much effort/some effort/no effort. For analysis we combined the options impossible/only with help of somebody else/much effort
Falls	Single question: ‘Have you been fallen once or more in the past six months?’ Yes/no [26]
Continence	Single question: ‘Did you use incontinence materials?’ Yes/no
Health	
Comorbidity	Charlson Comorbidity Index [27], score based on medical record
Polypharmacy	Single question: ‘Did you use five or more medications at home?’ Yes/no
Prior hospital use	Admission to hospital in the last six months
Depression	Two-question case-finding instrument. Score 1 or more [28]
Number of symptoms (physical and psychological)	Rotterdam Symptom Checklist (RSCL) [29] originally, the symptoms are on a four-point Likert scale, but we dichotomised the symptoms into present or absent on admission day. Factor analysis revealed the same two factors, namely physical and psychological symptoms
Self-rated health (two weeks before admission and on day of interview during admission)	EQ-5D VAS (0-100) [20]
Pain (on moment of interview, during admission)	Numeric rating scale (NRS) 0: no pain at all—10: the worst imaginable pain
Nutrition	Having either lost weight unintentionally and/or both having experienced a decreased appetite as well as used supplemental drinks or tube feeding [26]
Characteristics of admission	
Hospital length of stay	Days
Admission type	Acute/elective, derived from medical record
Specialism	Medical/surgical/intervention cardiology
Characteristics of goals	
Confidence in goals	NRS per goal 0: no confidence at all—10: full confidence. When a participant had more goals, the mean of the confidence ratings was calculated
Goal category	Open goals were coded as wanting to know what the matter is/controlling disease/staying alive/improving condition/alleviating complaints/daily functioning/social functioning/resuming work, hobbies/autonomy [5]. For analysis the categories wanting to know what the matter is/controlling disease were combined into category ‘disease’, improving condition/alleviating complaints were combined into category ‘complaints’, daily functioning/autonomy were combined into category ‘functioning’, social functioning/resuming work, hobbies were combined into category ‘social’

### Analysis

To assess correlations between PROMs and the anchor question, the Spearman’s rank order correlation was calculated. Correlations were interpreted according to Cohen’s criteria: 0.10 = small, 0.30 = medium, and 0.50 = large [30].

Associations between predicting factors and PROM were analysed by logistic regressions. First univariate logistic regression analyses were performed. Predictors with a *p* value ≤ 0.25 were then entered into a multivariate model [31]. Forward stepwise logistic regression model was used (likelihood ratio method, probability entry: 0.05, removal 0.10). Analyses were performed using IBM SPSS version 27. Pairwise deletion was used for all analyses to handle missing values.

## Results

**Fig. 1:**
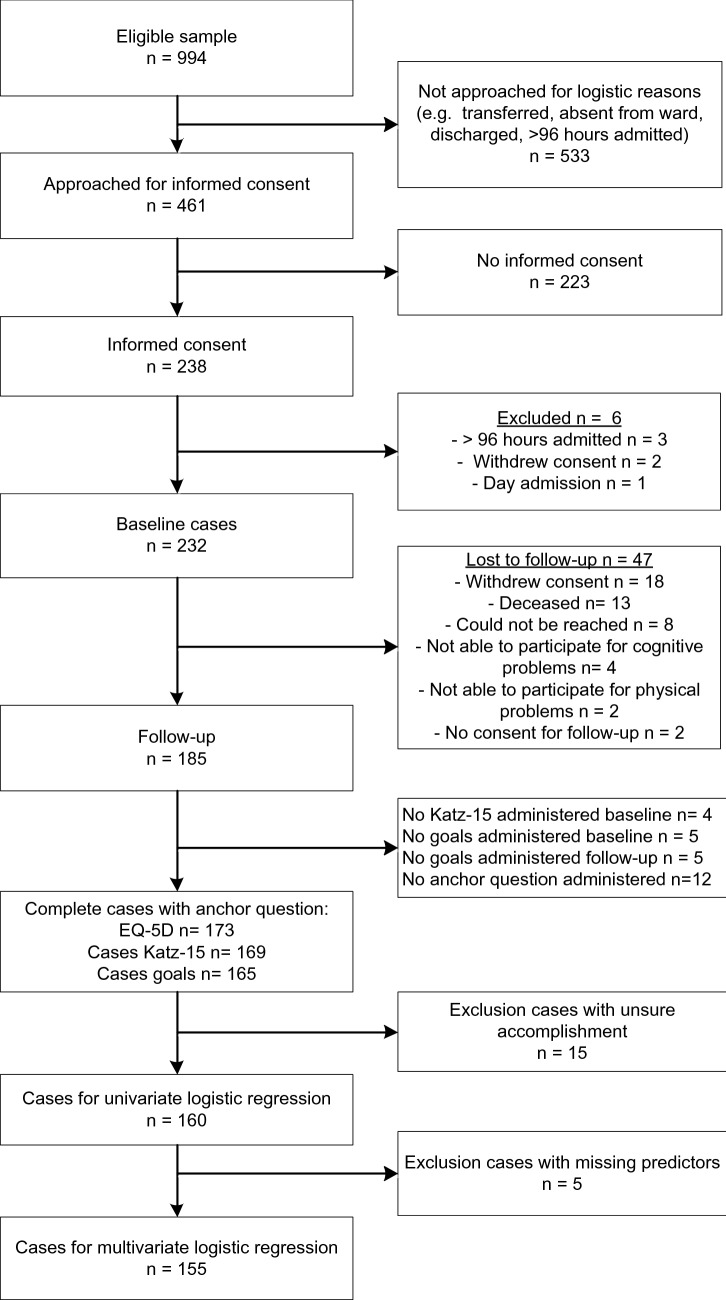
Flow diagram inclusion and exclusion of participants

Full details on the inclusion and exclusion of participants are shown in Fig. 1. We included 232 patients at baseline and 185 had a follow-up. As not all cases were complete, we had 155 to 173 cases for analysis. Reasons for not completing the baseline interview were: premature ending the interview because the participant had to leave for, for example, investigation or discharge, or in four cases the participant was unable to mention a goal.

During follow-up, goal accomplishment was not answered for 18 goals. Half of the time the participant did not know the answer. As part of the follow-up took place during the corona pandemic, accomplishment of eight goals could not be answered due to the then applying measures such as travel restrictions and closing of sport accommodations and restaurants. The goals were for example going on holiday, out for lunch, or to the sport club. Descriptive statistics of the sample are shown in Table 2, outcome measures and anchor question are shown in Table 3, and full details of the predictors are shown in Supplementary Information. We compared the participants with and without follow-up and found no significant differences, except for quality life, which was slightly better for participants with follow-up (Mann–Whitney *U* test: mean rank 94.40 versus 114.56, *p* = 0.05).

**Table 2:** Descriptive statistics sample (*n* = 185)

	*n* (%)
Age (years), median (IQR), range	75 (72–80), 70–98
Gender, male	109 (59)
Living situation	
Independent	180 (97)
Sheltered	2 (1)
Senior home	3 (2)
Nursing home	0
Marital status with partner	126 (68)
Admission type—acute	87 (49)
Missing	6
Specialism	
Medical	81 (45)
Surgical	44 (25)
Intervention cardiology	54 (30)
Missing	6

Goal category	*n* (%)
Matter	10 (3%)
Disease	60 (21%)
Alive	18 (6%)
Condition	21 (7%)
Complaints	44 (15%)
Daily function	42 (14%)
Social	20 (7%)
Work/hobbies	44 (15%)
Autonomy	8 (3%)
Other	24 (8%)
Missing	5

*IQR* interquartile range

**Table 3:**
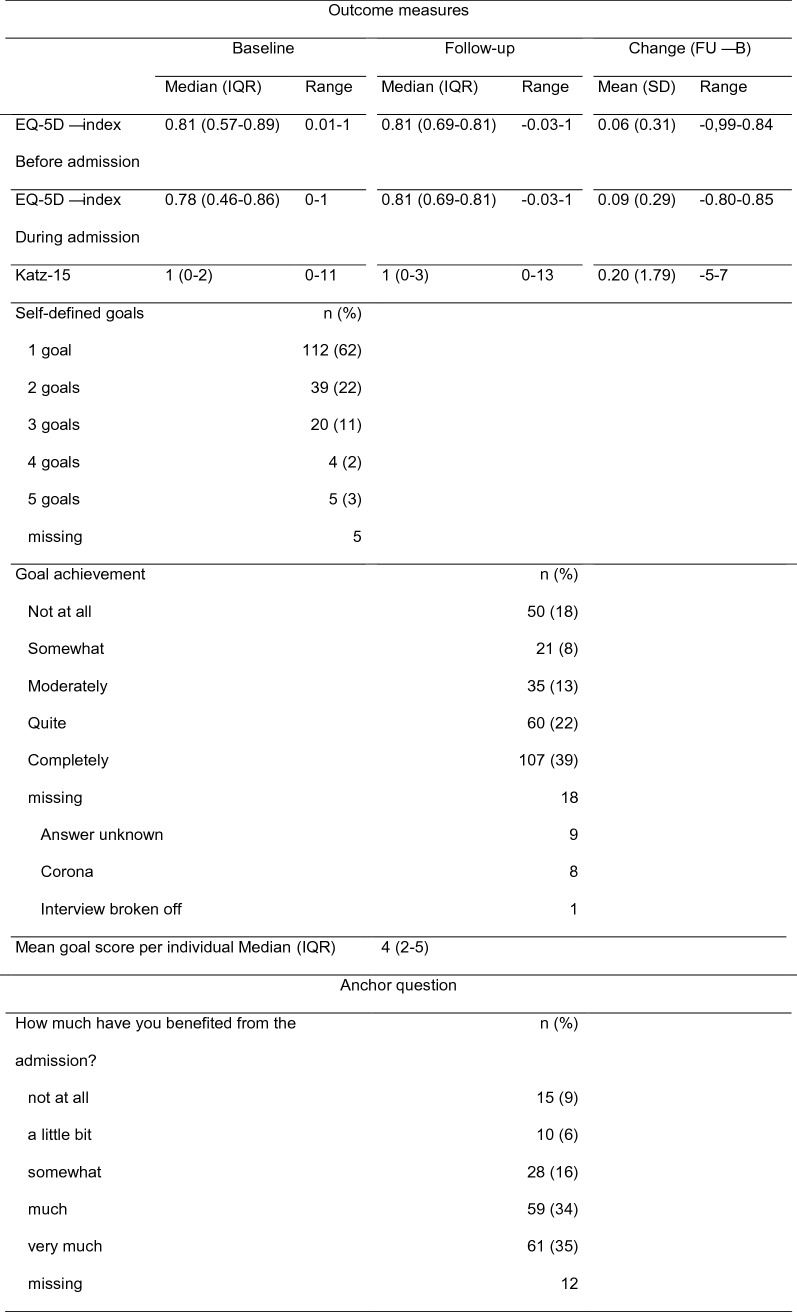
Descriptive statistics outcome measures and anchor question (*n* = 185)

### Correlations between anchor question and outcome measures

Table 4 shows the correlations between PROMs and the anchor question. The EQ-5D score comparing follow-up and situation before admission showed no correlation with the anchor question, while the EQ-5D score comparing follow-up and situation during admission and the Katz-15 difference score showed a small correlation. Accomplishment of self-defined goals showed a large correlation with the anchor question and was the only statistically significant correlation.

**Table 4:** Spearman’s rank order correlations between anchor question and diverse outcome measures

Anchor question	Difference score EQ-5D follow-up—before admission *n* = 173	Difference score EQ-5D follow-up—during admission *n* = 173	Difference score Katz-15 *n* = 169	Accomplishment self-defined goals *n* = 165
How much have you benefited from the admission?	0.00 (*p* = 0.97)	0.14 (*p* = 0.07)	− 0.13 (*p* = 0.09)	0.52 (*p* < 0.001)

### Regression analyses between self-defined goals and predictors

The PROM accomplishment of self-defined goals was dichotomised as follows: the answer options ‘not at all’, ‘somewhat’, and ‘moderately’ were defined as ‘not accomplished’; the answer options ‘quite’ and ‘completely’ were defined as ‘accomplished’. When a participant had more than one goal and all goals were attained from ‘not at all’ to ‘moderately’, it was defined as ‘not accomplished’; when all goals were attained as ‘moderately’ to ‘completely’, this was defined as ‘accomplished’. When participants had a combination of one or more goals attained as ‘not at all’ or ‘somewhat’ and ‘quite’ or ‘completely’, the definition of accomplishment was unsure, and therefore the case was removed. After dichotomisation of goal accomplishment, 101 cases were defined as ‘accomplished’ and 59 as ‘not accomplished’. We had to remove 15 cases because accomplishment was unsure. Cases that were removed had: a higher proportion living with partner (93% versus 66%, *χ*^2^ = 4.67, *p* = 0.02); more dependencies on the Katz-15 (Mann–Whitney *U* test: mean rank 116.80 versus 84.17, *p* = 0.01); and a higher proportion of goals in the category ‘social’ (87% versus 18%, *χ*^2^ = 35.32, *p* = 0.00).

Results of the univariate logistic regression analyses are shown in Table 5. The predictors gender, baseline quality of life, mobility, depression, self-rated health before admission, confidence in goals, goal category disease, and goal category complaints predicted goal achievement with statistical significance.

**Table 5:** Univariate and multivariate logistic regression for goal accomplishment

	Univariate (*n* = 160)		Multivariate (*n* = 155)	
	Odds ratio (95% CI)	*p* value	Odds ratio (95% CI)	*p* value
Age	1.04 (0.97–1.11)	0.27		
Gender-male	2.44 (1.26–4.72)	0.01	2.44 (1.15–5.16)	0.02
Marital status—with partner	0.99 (0.50–1.95)	0.98		
Health locus of control				
Internal	1.01 (0.94–1.08)	0.88		
Powerful others	1.02 (0.96–1.09)	0.44		
Chance	0.98 (0.92–1.05)	0.64		
Quality of life		0.00		0.05
Bad/reasonable	Reference		Reference	
Good	2.94 (1.01–8.56)	0.05	1.90 (0.56–6.43)	0.30
Very good/excellent	7.27 (2.22–23.80)	0.001	4.54 (1.19–17.26)	0.03
Katz ADL	0.89 (0.76–1.05)	0.16		
Cognitive functioning		0.54		
Could not reach January	Reference			
Could reach January with errors	0.55 (0.10–3.03)	0.49		
No errors	0.44 (0.09–2.19)	0.31		
Social functioning		0.13		
None of the time	Reference			
A little of the time	0.68 (0.24–1.88)	0.46		
Some of the time	0.36 (0.16–0.82)	0.02		
Most of the time	0.36 (0.12–1.06)	0.06		
All of the time	0.54 (0.14–2.16)	0.39		
Hearing—well	0.71 (0.30–1.69)	0.44		
Mobility—able to walk for 5 min		0.04		
No effort	Reference			
Some effort	0.39 (0.18–0.83)	0.02		
Much effort—impossible	0.48 (0.19–1.21)	0.12		
Falls—yes	1.29 (0.61–2.75)	0.51		
Incontinence	0.68 (0.30–1.54)	0.36		
Charlson Comorbidity	0.99 (0.86–1.14)	0.86		
Polypharmacy	0.78 (0.39–1.55)	0.47		
Prior hospitalisation	1.20 (0.61–2.37)	0.59		
Depression score	0.49 (0.25–0.98)	0.04		
Symptoms—physical	0.92 (0.83–1.02)	0.10		
Symptoms—mental	0.83 (0.69–1.01)	0.06		
Self-rated health 2 weeks before admission	1.03 (1.01–1.04)	0.01		
Self-rated health during admission	1.01 (0.99–1.03)	0.28		
Pain-NRS	1.02 (0.90–1.16)	0.80		
Nutrition—score yes	0.92 (0.46–1.85)	0.81		
Hospital length of stay	1.01 (0.94–1.08)	0.79		
Admission type—acute	0.86 (0.45–1.65)	0.65		
Speciality of admission		0.42		
Medical	Reference			
Surgical	1.27 (0.56–2.88)	0.57		
Intervention cardiology	1.22 (0.57–2.61)	0.62		
Confidence in goal achievement	1.52 (1.18–1.95)	0.00	1.43 (1.08–1.88)	0.01
Goal category disease	2.42 (1.16–5.03)	0.02		
Goal category complaints	0.27 (0.13–0.53)	0.00	0.26 (0.12–0.56)	0.00
Goal category social	1.67 (0.69–4.06)	0.26		
Goal category functioning	1.06 (0.50–2.24)	0.88		

Multivariate model: Nagelkerke *R*^2^ = 0.29; *χ*^2^ = 37.67, *p* < 0.001

The predictors gender, baseline quality of life, (I)ADL, social functioning, mobility, depression, number of physical and psychological symptoms, self-rated health before admission, confidence in goals, goal category disease, and goal category alleviating complaints were entered into a multivariate analysis. Since five participants had one or more missing values in these predictors, we had 98 cases defined as ‘accomplished’ and 57 as ‘not accomplished’ for multivariate analysis. As shown in the last two columns of Table 5, the final model had four predictors, indicating that being a man and having a higher confidence in goals increased the odds for goal accomplishment and having goals in the category alleviating complaints reduced the odds for goal accomplishment. From baseline quality of life, only the odds of good/excellent quality of life were significant when compared to bad/reasonable.

## Discussion

The first aim of this study was to assess which PROM is best suited to evaluate patient-relevant outcomes of hospitalisation. Therefore, multiple PROMs were compared with the anchor question whether participants experienced benefit from their hospitalisation. When comparing this perceived benefit with change in a frequently used general quality of life instrument, the EQ-5D, and change in daily functioning with the Katz-15, we noticed no, or only a small non-significant correlation. In contrast, accomplishment of self-defined goals had a large correlation with experienced benefit from hospitalisation.

Most participants did not change in EQ-5D score at all. This could be caused by a lack of responsiveness of the EQ-5D. Ceiling effects are described, as well as too little answer options [32–34]. Ceiling effects were also present in our study, with 16–20% scoring 1 at baseline. A review of reviews showed that the performance of the EQ-5D is inconsistent between different conditions, it appeared to be responsive for many diseases, but for others it was not [35]. This could be another explanation, since we had a heterogeneous group of participants. Moreover, responsiveness was mostly based on effect sizes or standardised response means [35]. This method is debatable, since these measures reflect the magnitude of change, but not the validity of change [36]. As the change, or lack of change, did not correlate with our anchor question, it apparently did not correspond with what these participants considered as a relevant (lack of) benefit.

Another debate is the relevance of the items of the EQ-5D. When comparing the goals with the domains of the EQ-5D, we saw that 90 participants had goal(s) that could not be linked to an EQ-5D domain, for 68 participants their goal(s) were represented in one domain and for 7 participants in two domains. No participants had goals represented by three or more domains. The most prevalent domain (46 times) was ‘usual activities’, followed by mobility (24×). Apparently, the EQ-5D does not reflect the specific relevant outcomes of hospitalisation for individual patients.

The correlation of the change in Katz-15 with our anchor question was also small and non-significant. Daily functioning is often mentioned as an important outcome for older persons [5, 37–40]. In our sample 22% of the participants mentioned at least one goal coded as daily functioning, which endorses the importance of daily functioning. However, this also means that 78% of the participants had no goals related to daily functioning. So, using daily functioning as an outcome of hospitalisation does not represent the diversity of personal goals.

Another explanation for the difference in strengths of correlations could be the time course direction of the questions. Both the anchor question and goal achievement ask participants to look back during follow-up to assess how much they have benefited or whether they have achieved their goals, while for the EQ-5D and Katz-15 difference scores are computed between follow-up and baseline. Therefore it is not surprising that the correlation between goals and anchor question is stronger. However, the magnitude of the difference is so large that we conclude that accomplishment of individually stated goals best represents the benefit experienced by individual participants, because goals are very individual.

From the variables predicting goal accomplishment, our second aim, it seems that more subjective indicators of health and functioning, such as quality of life, depressive symptoms, and self-rated health, are better predictors than more objective predictors, such as ADL functioning, falling, comorbidity, and nutritional status. Since goal accomplishment is also subjective, common variance may be shared. However, relationships between subjective PROMs and objective outcomes are common, such as survival [41]. The importance of subjective predictors means that medical decision-making should not only be based on medical indicators, but the input of the individual patient is at least as important. Subjective quality of life, goals, and confidence should be discussed and weighted to reach shared decision.

The importance of confidence in goal achievement can be explained from the construct of hope. Hope is always related to a goal (something for which to hope) and involves thoughts about a strategy for achieving goals and the ability to begin and continue the selected strategy [42]. Hope and optimism are also described as predictors for positive outcomes, both subjective and objective outcomes [42–46]. Although a quite new area, there are some promising interventions described to enhance hope, such as cognitive coping techniques or “hope therapy”, where goal setting has a prominent place [42]. Further research is needed to investigate whether these interventions also promote goal accomplishment.

The influence of gender was surprising. An explanation could be that men in our sample had less physical and mental symptoms, less limitations in (I)ADL and less mobility problems. This might be a reflection of the evidence that a higher proportion of older European women are in a frail state [47].

The odds ratios for the goal categories ‘Disease’ and ‘Complaints’ were contradicting, indicating that, according to participant experience, the hospital is successful in diagnosing and restoring disease-specific problems, such as removing tumours, restoring sinus rhythm or improving kidney function, but less successful in ameliorating the symptoms patients experience, such as fatigue, shortness of breath, or pain. Therefore, during hospitalisation more attention is needed for symptom experience and relief of complaints and not only for objective outcomes. Better follow-up care may be needed to continue symptom management after discharge, but maybe also patient expectations towards therapy need to be tempered. For example, fatigue more often worsens after hospitalisation than improves [48].

The final model contained four variables, namely gender, quality of life, confidence in goal achievement, and goal category complaints. The Nagelkerke *R*^2^ is a measure of overall effect size used for logistic regression. It runs from 0 to 1, and its interpretation is comparable with *R*^2^ in multiple regression analysis [49, 50]. The Nagelkerke *R*^2^ of our final model was 0.29, so much of the variance was not explained by the variables. It could be that we were missing variables, but it is also possible that goal accomplishment is very individual and therefore difficult to predict by statistical models.

### Strengths and limitations

The strengths of our study are that we highlighted the importance of individualised goals and outcomes, instead of ‘one outcome fits all’. By asking open questions, the individual patient perspective was disclosed. Due to a heterogeneous sample, we have high external validity.

However, also some limitations were present. First, we had no information about the importance of the goals, while the importance of goals can influence the perceived benefit when goals are (not) accomplished [11]. The importance of goals is especially relevant when, within one participant, some goals are accomplished and others not. This was the reason we left out 15 participants with contradicting results, but we do not know what the effect of this exclusion was.

Since the 15 excluded cases, per definition, all had two or more goals, we had not included the predictor ‘number of goals’, because this would give a biased result. As a result, we cannot draw any conclusions about a potential effect of the number of indicated goals.

Our sample also had some limitations: almost all participants lived independently and we could not include any nursing home residents. Therefore, an impact of being a nursing home resident on goal accomplishment is unknown. Due to our inclusion criteria, we had little variance in cognition and therefore we cannot draw conclusions about an effect of cognition. Finally, our sample was not large enough for cross-validation. Therefore, caution is needed to draw conclusions on a stepwise regression model.

### Conclusions and recommendations

Goals of hospitalisation are very individual and therefore the experienced benefit is better represented by accomplishment of individually stated goals then generalised PROMs. The accomplishment of goals is predicted by subjective indicators such as quality of life and confidence in goal achievement, and also whether goals were disease specific or ameliorating complaints. Therefore, medical decision-making should not only be based on medical indicators, but also the input of the patient should be seen as at least as important, especially confidence should be discussed. More attention for symptom management is needed during and after hospitalisation. Further research is recommended to investigate whether hope-enhancing interventions also enhance goal accomplishment.

## Supplementary Information

Below is the link to the electronic supplementary material.

Supplementary file1 (DOCX 27 KB)
