# Supplementary material for: Goal-based outcomes of hospitalisation of older adults are predicted by gender, confidence, quality of life and type of goals
Source: Eur Geriatr Med. 2022 Oct 7;13(6):1377–89. doi: 10.1007/s41999-022-00698-2 (PMC9722898; doi:10.1007/s41999-022-00698-2)
Supplement: Supplementary file 1 — Supplementary file1 (DOCX 27 KB) [file 41999_2022_698_MOESM1_ESM.docx]

**Supplementary Information. Descriptive statistics predictors**

**Article title:** Goal-based outcomes of hospitalisation of older adults are predicted by gender, confidence, quality of life and type of goals

**Journal name:** Europan Gericatric Medicine

**Author names:** Maria Johanna van der Kluit, Sanne Tent, Geke J. Dijkstra, Sophia E. de Rooij

**Corresponding author:** Maria Johanna van der Kluit, University of Groningen, University Medical Center Groningen, University Center for Geriatric Medicine, m.j.van.der.kluit@umcg.nl

(n=185)

|  | n (%) |
| --- | --- |
| Age (years), median (IQR), range | 75 (72-80), 70-98 |
| Gender - male | 109 (59) |
| Living situation  Independent  Sheltered  Senior home  Nursing home | 180 (97)  2 (1)  3 (2)  0 |
| Marital status  with partner | 126 (68) |
| Health locus of control, mean (SD), range  Internal  Powerful others  Chance  Missing (n) | 21.02 (4.93), 10-34  21.33 (5.39), 8-32  21.12 (5.06), 8-35  7-12 |
| Baseline Quality of life  Bad  Reasonable  Good  Very good  Excellent  Missing | 3 (2)  19 (10)  104 (56)  41 (22)  15 (8)  3 |
| (I)ADL: Katz-15, median (IQR), range  Missing (n) | 1 (0-2), 0-11  4 |

| Cognitive functioning: Months backward test  Subject engages with testing procedures  Subject successfully shifts to backwards testing  Subject can reach July  Subject can reach January  If the subject makes any errors, they recognize this  Completion of the test takes less than 60 seconds  missing | 1 (1)  4 (2)  6 (3)  30 (16)  14 (8)  125 (68)  5 |
| --- | --- |
| Social functioning: interfered with social activities  none of the time  a little of the time  some of the time  most of the time  all of the time  missing | 72 (40)  26 (14)  50 (28)  20 (11)  13 (7)  4 |
| Hearing - well  missing | 151 (82)  3 |
| Mobility: able to walk for 5 minutes  Impossible  Only with help of somebody else  Much effort  Some effort  No effort  missing | 13 (7)  1 (1)  15 (8)  50 (27)  103 (57)  3 |
| Falls - yes | 45 (24) |
| Continence: using incontinence materials  missing | 34 (19)  4 |
| Charlson Comorbidity, median (IQR), range  missing | 3 (2-4), 0-10  6 |
| Polypharmacy: using > 5 medications  missing | 119 (66)  5 |
| Prior hospitalization in last six months  missing | 70 (39)  5 |
| Depression score  missing | 60 (33)  4 |
| Symptoms physical, median (IQR), range  Missing (n) | 5 (2-7), 0-16  1 |
| Symptoms psychological, median (IQR), range  Missing (n) | 1 (0-3), 0-8  1 |
| Self-rated health two weeks before admission, mean (SD), range | 63.90 (18.82), 16-100 |
| Self-rated health during admission, mean (SD), range | 61.56 (18.02), 0-100 |
| Pain-NRS, median (IQR), range  Missing (n) | 1 (0-4), 0-8  4 |
| Nutrition- score yes | 40 (22) |
| Hospital length of stay, median (IQR), range | 4 (3-7), 1-28 |
| Admission type – acute  missing | 87 (49)  6 |
| Specialism  Medical  Surgical  Intervention cardiology  missing | 81 (45)  44 (25)  54 (30)  6 |
| Confidence in goals, median (IQR), range | 8 (7-9), 3.5-10 |

| Goal category  matter  disease  alive  condition  complaints  daily function  social  work/hobbies  autonomy  other  missing | n (%)  10 (3%)  60 (21%)  18 (6%)  21 (7%)  44 (15%)  42 (14%)  20 (7%)  44 (15%)  8 (3%)  24 (8%)  5 |
| --- | --- |

IQR=Interquartile range, SD=Standard deviation (I)ADL = (instrumental) activities of daily living, NRS=Numeric rating scale
